# Inflammatory related plasma proteins involved in acute preschool wheeze

**DOI:** 10.1002/clt2.12308

**Published:** 2023-11-01

**Authors:** Idun Holmdahl, Sandip Chakraborty, Angela Hoyer, Anastasia Filiou, Anna Asarnoj, Anders Sjölander, Magnus P. Borres, Marianne van Hage, Gunilla Hedlin, Jon R. Konradsen, Cilla Söderhäll

**Affiliations:** ^1^ Department of Women's and Children's Health Karolinska Institutet Stockholm Sweden; ^2^ Astrid Lindgren's Children's Hospital Karolinska University Hospital Stockholm Sweden; ^3^ Thermo Fisher Scientific Uppsala Sweden; ^4^ Department of Women's and Children's Health Uppsala University Uppsala Sweden; ^5^ Division of Immunology and Allergy Department of Medicine Solna Karolinska Institutet and Karolinska University Hospital Stockholm Sweden

**Keywords:** asthma, inflammation, olink, plasma proteins, preschool wheeze

## Abstract

**Background:**

Preschool wheeze is a risk factor for asthma development. However, the molecular mechanism behind a wheezing episode is not well understood.

**Objective:**

Our aims were to assess the association of plasma proteins with acute preschool wheeze and to study the proteins with differential expression at the acute phase at revisit after 3 months. Additionally, to investigate the relationship between protein expression and clinical parameters.

**Method:**

We measured 92 inflammatory proteins in plasma and clinical parameters from 145 children during an episode of preschool wheeze (PW) and at the revisit after 3 months (PW‐R, *n* = 113/145) and 101 healthy controls (HC) aged 6–48 months in the GEWAC cohort using the antibody‐mediated proximity extension‐based assay (Olink Proteomics, Uppsala).

**Results:**

Of the 74 analysed proteins, 52 were differentially expressed between PW and HC. The expression profiles of the top 10 proteins, Oncostatin M (OSM), IL‐10, IL‐6, Fibroblast growth factor 21 (FGF21), AXIN1, CXCL10, SIRT2, TNFSF11, Tumour necrosis factor β (TNF‐β) and CASP8, could almost entirely separate PW from HC. Five out of 10 proteins were associated with intake of oral corticosteroids (OCS) 24 h preceding blood sampling (OSM, CASP8, IL‐10, TNF‐β and CXCL10). No differences in protein expression were seen between PWs with or without OCS in comparison to HC. At the revisit after 3 months, differential protein expressions were still seen between PW‐R and HC for three (IL‐10, SIRT2 and FGF21) of the 10 proteins.

**Conclusion:**

Our results contribute to unravelling potential immunopathological pathways shared between preschool wheeze and asthma.

## 1 INTRODUCTION

Preschool wheeze is a well‐known risk factor for the development of asthma and affects one third of all children below 3 years of age and half of the children under the age of 6.^1^, ^2^, ^3^, ^4^ Wheezing episodes often lead to hospital admissions^2^, ^4^ and are most commonly triggered by viral infections, with rhinovirus (RV) being one of the most frequent pathogens. Different phenotypes of preschool wheeze have been proposed, but they are not stable over time and therefore their clinical value is uncertain.^5^ It has been suggested that cytokine responses to viral infections in the acute wheezing episodes early in life may be of importance in the development of recurrent wheeze and, in continuation, allergic asthma.^6^ Many children with preschool wheeze grow out of their symptoms by school age and do not develop asthma, but they may still have residual lung function deficits,^7^ suggesting structural changes during previous wheezing episodes. Thus, further characterization of the inflammatory processes involved in preschool wheeze may lead to improved understanding of preschool wheeze as a precursor of asthma.

Impaired interferon (IFN) responses have been reported among children with wheeze and asthma. Some studies have shown a decreased level of IFN response to respiratory viruses^8^, ^9^ leading to impaired viral control and a more severe disease, while others have shown an increased IFN response.^10^, ^11^ Inappropriate activation of the type 2 (T2) inflammatory cascade in response to viral infection, especially RV infections, is associated with asthma inception and exacerbation.^12^ Exaggerated T2 responses have been shown to be important in school‐age asthma, but relatively little is known about the immunopathology of preschool wheeze.^13^

We hypothesized that a distinct pattern of inflammatory proteins in plasma can be observed during an episode of preschool wheeze and that this pattern could contribute to the understanding of preschool wheeze as a precursor of asthma. Our primary aim was to compare the levels of inflammation‐related proteins obtained from preschool children during an acute wheezing episode and from healthy controls. Secondarily, we aimed to study whether the observed differences at inclusion persisted at a revisit 3 months later. Thirdly, we aimed to investigate the relationship between inflammatory related proteins in plasma and clinical parameters.

## 2 MATERIAL AND METHOD

### 2.1 Study population

Children (*n* = 156, 6–48 months of age) included in the GEWAC (Gene Expression in Wheezing and Asthmatic Children) study were recruited from the emergency department at Astrid Lindgren's Children's Hospital between 2008 and 2012 when presenting with symptoms of preschool wheeze. During the same period, 102 age‐matched healthy controls were recruited from the surgical day‐care ward at Astrid Lindgren's Children's Hospital in Stockholm, Sweden, as previously described.^14^ Inclusion and exclusion criteria are given in Table S1. Children with preschool wheeze came to a revisit after approximately 3 months (*n* = 130).

**FIGURE 1:**
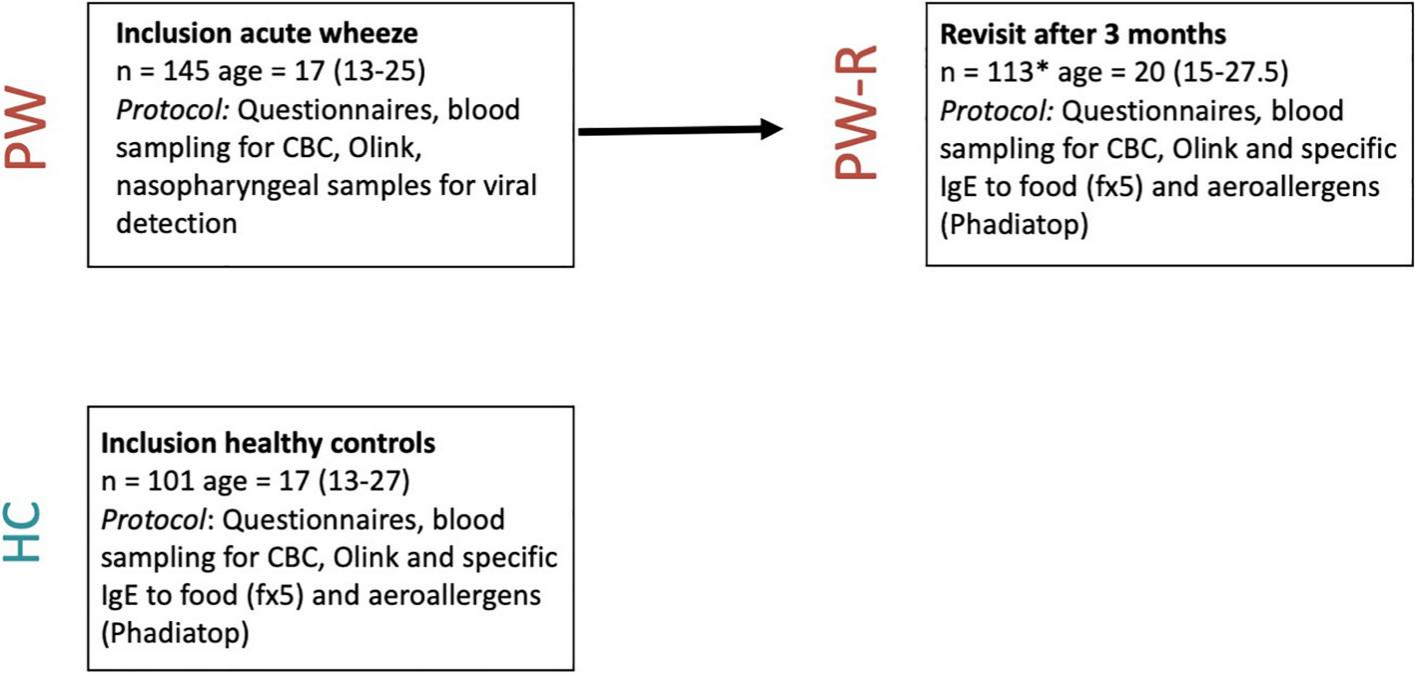
Flow‐chart of number of children with an acute episode of preschool wheeze (PW) and healthy controls (HC) that attended the different follow‐ups and sample collections at each visit. Age in months presented as medians with inter‐quartile ranges (IQR). Comparisons are made between PW at inclusion (during an acute wheezing episode) and HC at inclusion as well as between PW at revisit after 3 months (PW‐R) and HC at inclusion. *113 who left plasma at revisit. CBC, Complete blood count.

In this study, 145 children recruited during an acute episode of preschool wheeze (PW) and 101 healthy controls (HC) with plasma available at inclusion were included. A cross‐sectional comparison of protein expression was made between PW and HC at inclusion. Protein expression among PW with plasma available at the revisit after 3 months (PW‐R, *n* = 113) in comparison to HC with plasma available at inclusion (*n* = 101), was also studied (Figure 1).

### 2.2 Sample collection

The study protocol, previously described,^3^ included standardized questionnaires regarding demography, breastfeeding, exposure to tobacco smoke, preschool attendance, parental asthma and allergies, previous infections and wheeze, current atopic and asthmatic symptoms and use of asthma medication. Nasopharyngeal swabs for viral detection were taken at the emergency visit and analysed as previously described.^14^ Doctors’ diagnoses of asthma at inclusion and revisit are based on national guidelines developed by the Swedish paediatric society and are based on the number of wheezing episodes and history of atopy.^15^ Blood samples were collected and analysed for complete blood count, vitamin D and allergen‐specific IgE antibodies against a mix of common food allergens, fx5 (milk, egg white, wheat, codfish, peanut and soya bean) and a mix of common airborne allergens, Phadiatop (house dust mite, cat, horse, dog, timothy, birch, and mugwort, *Cladosporium herbarum*) (Thermo Fisher Scientific, Uppsala, Sweden), as previously described.^3^ Allergic sensitization was defined as allergen‐specific IgE≥0.35 kU_A_/L.

### 2.3 Proteomic analyses

Blood samples were collected at inclusion and at the revisit after 3 months from PW and at inclusion from HC. EDTA plasma was collected in a standardized way and stored at −80°C until analysis. Ninety‐two immune‐related proteins were measured with the Olink Target 96 Inflammation panel using the antibody‐mediated proximity extension technology^16^ (Olink Proteomics, Uppsala, Sweden).

### 2.4 Statistical analyses

Data from the Olink analysis were obtained as Normalized Protein eXpression (NPX) values; these are log_2_ transformed normalized values. Proteins for which more than 15% of the samples had values below the Limit of Detection (LOD) were excluded from the analysis (Table S2). Based on this, 18 of the 92 proteins were excluded during quality control. NPX values were converted into a linear scale (2^(NPX)^) and generalized linear model design = ∼ Sample‐type (AW and HC) was used to calculate differential protein expression by the edgeR package.^17^ Log_2_ transformed fold change and False Discovery rate (FDR) by the Benjamini‐Hochberg method^18^ were calculated for each protein. The top 10 significant differentially expressed proteins between PW and HC with abs(log_2_ fold change) > 1, FDR <0.05 were selected for further analysis. A heatmap with unsupervised clustering of the top 10 differentially expressed proteins in the 246 samples was plotted using their NPX value with the scaled row.

Descriptive statistics were performed using SPSS version 27 (IBM). A two‐tailed probability of <0.05 was considered statistically significant. The Chi‐square test was used to examine proportional differences between the two groups and Fisher's exact test was used on small sample sizes. Continuous variables with normal distribution were examined using an unpaired *t*‐test and skewed variables were examined with the non‐parametric test, Mann‐Whitney *U* test. The correction for multiple testing was performed using Bonferroni's correction and adjusted *p*‐values are presented in Tables 1 and 2, along with unadjusted *p*‐values.

**TABLE 1:** Baseline characteristics of children with an acute episode of preschool wheeze (PW) and healthy controls (HC) at inclusion and children with preschool wheeze at revisit, 3 months after an episode of acute wheeze (PW‐R) and HC at inclusion.

Variables	PW *n* = 145	HC *n* = 101	*p*‐value	Adjusted *p*‐value^a^	PW‐R *n* = 113	HC *n* = 101	*p*‐value	Adjusted *p*‐value^a^
Age in months at inclusion, median (IQR)	17 (13–25)	17 (13–27)	0.35	1	17 (12–24.5)	17 (13–27)	0.31	1
Age in months, revisit, median (IQR)					20 (15–27.5)	17 (13–27)	0.095	1
Male sex, *n* (%)	97 (66.9)	79 (78.2)	0.053	0.48	75 (66.4)	79 (78.2)	0.054	0.65
Parental asthma/allergy, *n* (%)	98 (73.1)	46 (46.9)	**<0.001**	**<0.001**	83 (74.1)	46 (46.9)	**<0.001**	**<0.001**
>6 RTIs^b^/year, *n* (%)	85 (64.4)	19 (19.2)	**<0.001**	**<0.001**	73 (66.4)	19 (19.2)	**<0.001**	**<0.001**
LTRA^c^ at inclusion, *n* (%)	13 (9.0)				11 (9.7)			
ICS^d^ at inclusion, *n* (%)	59 (40.7)				46 (40.7)			
OCS^e^ within 24 h of blood sampling at inclusion, *n* (%)	111 (76.6)				85 (75.2)			
LTRA at revisit, *n* (%)					15 (13.4)			
ICS at revisit, (*n* %)					44 (39.3)			
OCS between inclusion and revisit, *n* (%)					14 (12.7)			
Fx5 positive, *n* (%)	27 (23.1)	16 (20.0)	0.61	1	26 (23.2)	16 (20.0)	0.59	1
Phadiatop positive, *n* (%)	9 (7.7)	3 (3.8)	0.37	1	9 (8.0)	3 (3.8)	0.23	1
Atopic dermatitis at inclusion, *n* (%)	26 (19.3)	7 (7.1)	**0.008**	0.07	23 (20.4)	7 (7.1)	**0.006**	0.07
First time wheeze at inclusion, *n* (%)	27 (22.1)				26 (23.0)			
Hospitalized at inclusion, *n* (%)	98 (80.3)				92 (81.4)			
RTIs^b^ at inclusion,^f^ *n* (%)	137 (94.5)				108 (95.6)			
Eosinophils at inclusion 10^9^/L, median (IQR)	0.01 (0.01–0.1)	0.2 (0.1–0.3)	**<0.001**	**<0.001**	0.05 (0.05–0.1)	0.2 (0.1–0.3)	**<0.001**	**<0.001**
Neutrophils at inclusion 10^9^/L, median (IQR)	7.5 (4.3–10.4)	2.6 (1.8–3.4)	**<0.001**	**<0.001**	7.1 (4.4–10.3)	2.6 (1.8–3.4)	**<0.001**	**<0.001**
Eosinophils at revisit 10^9^/L, median (IQR)					0.3 (0.2–0.6)	0.2 (0.1–0.3)	**<0.001**	**0.001**
Neutrophils at revisit 10^9^/L, median (IQR)					3.1 (2.1–4.3)	2.6 (1.8–3.4)	**0.013**	0.16

*Note*: Bold indicate *p* < 0.05.

^a^ Bonferroni adjusted *p*‐values.

^b^ Respiratory tract infections.

^c^ Leukotriene Receptor Antagonist.

^d^ Inhaled Corticosteroids.

^e^ Oral Corticosteroids.

^f^ All 8 who answered “No” had positive NPH samples (3 Bocavirus, 1 Parainfluenza, 3 Rhinovirus and 1 Respiratory Syncytial Virus).

**TABLE 2:** Baseline characteristics of children with an acute episode of preschool wheeze (PW) receiving oral corticosteroids (OCS) 24 h prior to blood sampling, at the acute wheezing episode, in comparison PW not receiving OCS.

Variables	PW with OCS *n* = 111	PW without OCS *n* = 34	*p*‐value	Adjusted *p*‐value^a^
Age in months at inclusion, median (IQR)	18 (12–25)	16 (11.8–23.8)	0.74	1
Male sex, *n* (%)	69 (62.2)	28 (82.4)	**0.029**	0.66
Parental asthma/allergy, *n* (%)	77 (75.5)	21 (65.6)	0.27	1
>6 RTIs^b^/year, *n* (%)	64 (64.0)	21 (65.6)	0.87	1
LTRA^c^ at inclusion, *n* (%)	8 (7.2)	5 (14.7)	0.18	1
ICS^d^ at inclusion, *n* (%)	45 (40.5)	14 (41.2)	0.95	1
LTRA at revisit, *n* (%)	12 (12.5)	4 (16.7)	0.74	1
ICS at revisit, *n* (%)	36 (37.5)	14 (58.3)	0.064	1
OCS^e^ between inclusion and revisit, *n* (%)	11 (11.6)	5 (21.7)	0.31	1
fx5 positive, *n* (%)	23 (25.3)	4 (15.4)	0.29	1
Phadiatop positive, *n* (%)	9 (9.9)	0 (0)	0.20	1
Atopic dermatitis at inclusion, *n* (%)	21 (20.4)	5 (15.6)	0.55	1
First time wheeze at inclusion, *n* (%)	19 (19.8)	8 (30.8)	0.23	1
Hospitalized at inclusion, *n* (%)	84 (87.5)	14 (53.8)	**<0.001**	**0.003**
Number of days admitted to hospital, median (IQR)	1 (1–2)	1 (0‐1.25)	**0.005**	0.12
Number of wheezing episodes between inclusion and revisit, median (IQR)	2 (0–3)	2 (0.75–3.25)	0.18	1
Attended the emergency department between inclusion and revisit, *n* (%)	58 (61.1)	15 (60.0)	0.92	1
Doctor's diagnosis of asthma at inclusion, *n* (%)	59 (53.2)	20 (58.8)	0.56	1
Doctor's diagnosis of asthma at revisit, *n* (%)	49 (51.0)	15 (57.7)	0.55	1
Eosinophils at inclusion 10^9^/L, median (IQR)	0.05 (0.05‐0.05)	0.1 (0.05‐0.4)	**<0.001**	**0.01**
Neutrophils at inclusion 10^9^/L, median (IQR)	7.7 (4.5–10.8)	6 (3.9–9.3)	0.17	1
Eosinophils at revisit 10^9^/L, median (IQR)	0.4 (0.2–0.6)	0.2 (0.2–0.4)	0.098	1
Neutrophils at revisit 10^9^/L, median (IQR)	3.2 (2.3–4.6)	3.0 (1.9–3.9)	0.44	1

^a^ Bonferroni adjusted *p*‐values.

^b^ Respiratory tract infections.

^c^ Leukotriene Receptor Antagonist.

^d^ Inhaled Corticosteroids.

^e^ Oral Corticosteroids.

### 2.5 Ethics

The study protocol was approved by the Regional Ethics Committee of Karolinska Institutet, Stockholm, Sweden (Dnr 2008/378‐31/4 and Dnr 2014/399‐31/3). Written informed consent was obtained from the legal guardians.

## 3 RESULTS

The study population consisted of 145 children recruited during an acute episode of preschool wheeze (PW) and 101 healthy controls (HC), aged 6–48 months at the time of inclusion to the GEWAC study. Among the included 145 PWs, 113 children came to a revisit after 3 months (PW‐R).

### 3.1 Baseline characteristics of preschool wheezers and healthy controls

Baseline characteristics of PW (*n* = 145) and HC (*n* = 101) are shown in Table 1. PW had more parental heredity for asthma and allergy (73.1% vs. 46.9%, *p* < 0.001) and more respiratory tract infections (RTIs) per year prior to inclusion (64.4% vs. 19.2%, *p* < 0.001). Hospitalization was needed for 80.3% of all PW at inclusion and 22.1% experienced their first episode of wheeze at inclusion. The comparison of baseline characteristics between PW‐R (*n* = 113) and HC (*n* = 101) showed similar results (Table 1). A dropout analysis was performed of PW that did not attend the revisit; no differences were seen in comparison to PW‐R (Table S4).

### 3.2 Proteomic differences between preschool wheezers and healthy controls at inclusion

**FIGURE 2:**
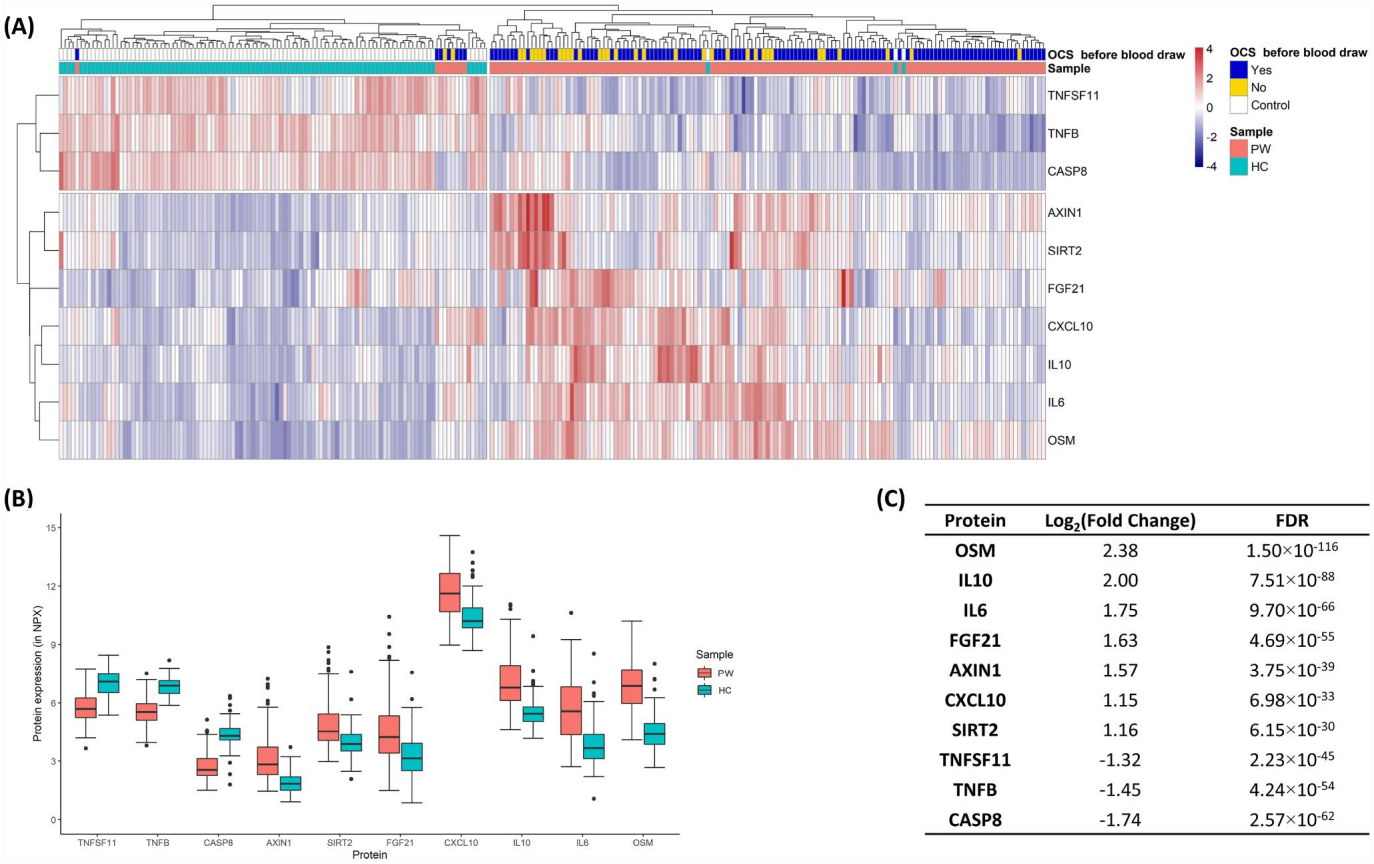
The top 10 most differentially expressed proteins between 145 children with an acute episode of preschool wheeze (PW) and 101 healthy controls (HC). (A) Heat map, plotted with unsupervised clustering, of the top 10 most differentially expressed proteins. (B) Box plot showing protein expression between PW and HC. (C) Fold change and false discovery rate (FDR) of the top 10 most differentially expressed proteins between PW and HC. AXIN 1, Axis inhibition protein 1; CASP8, Caspase 8; CXCL10, C‐X‐C motif chemokine ligand 10; FC, fold change; FDR, false discovery rate; FGF21, Fibroblast Growth Factor 21; HC, healthy controls; IL‐10, Interleukin 10; IL‐6, Interleukin 6; OCS, oral corticosteroids; OSM, Oncostatin M; PW, children with an acute episode of preschool wheeze; SIRT2, Sirtuin 2; TNFβ, Tumor necrosis factor β; TNFSF11, TNF superfamily 11.

Significant differences were seen between the PW and HC for 52 of the 74 analysed proteins (Table S3). With unsupervised clustering, the top 10 most differentially expressed proteins separated PW from HC, except for nine PW and three HC (Figure 2A). PW showed significantly higher expression of seven proteins (Oncostatin M (OSM), Interleukin 6 (IL‐6), Interleukin 10 (IL‐10), C‐X‐C motif chemokine ligand 10 (CXCL10), Sirtuin 2 (SIRT2), Fibroblast growth factor 21 (FGF21) and Axis inhibition protein 1 (AXIN1)), and lower levels of the remaining three proteins (Tumour necrosis factor β (TNF‐β), Caspase 8 (CASP8) and TNF superfamily member 11 (TNFSF11)), compared to the HC (Figure 2B,C and Table S5).

### 3.3 Characteristics of preschool wheezers that clustered among healthy controls

Nine of the PWs clustered among the HC with unsupervised clustering (Figure 2A). Comparing those nine PW with the rest of the PW (*n* = 136), higher blood‐eosinophil (0.2 × 10^9^/L vs. 0.05 × 10^9^/L, *p* = 0.001) and lower blood‐neutrophil (3.7 × 10^9^/L vs. 7.7 × 10^9^/L, *p* = 0.007) counts were observed (Table S6). The three HC that clustered among the PWs were all males, aged 12–28 months and specific IgE negative to fx5 and Phadiatop. Two of them had known parental heredity for asthma and allergy and more than six RTIs the year preceding inclusion.

### 3.4 The effect of oral corticosteroids on protein expression at inclusion

**FIGURE 3:**
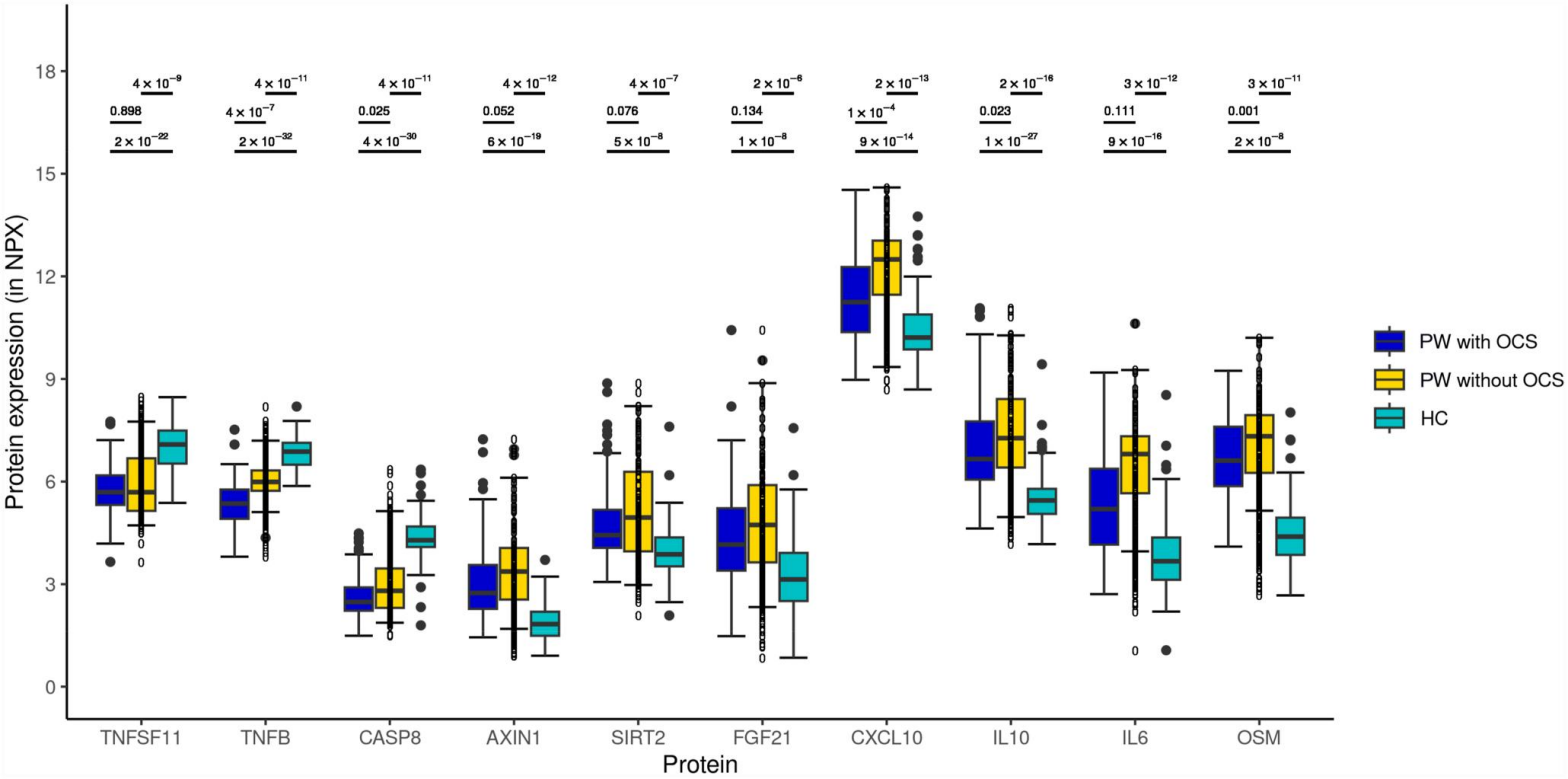
The box plot showing protein expression between children with an acute episode of preschool wheeze (PW) at inclusion, with and without oral corticosteroids (OCS) and healthy controls (HC). Statistical significance between the two groups was calculated using the two‐tailed Mann‐Whitney *U* test. AXIN1, Axis inhibition protein 1; CASP8, Caspase 8; CXCL10, C‐X‐C motif chemokine ligand 10; FGF21, Fibroblast growth factor 21; IL‐10, Interleukin 10; IL‐6, Interleukin 6; OCS, oral corticosteroids; OSM, Oncostatin M; SIRT2, Sirtuin 2; TNFSF11, TNF superfamily 11; TNFβ, Tumor necrosis factor β.

Comparing the samples from PW with (*n* = 111) and PW without (*n* = 34) oral corticosteroids (OCS) treatment 24 h preceding blood sampling, a significantly lower level of protein expression of five (OSM, CASP8, IL‐10, TNF‐β and CXCL10) of the top 10 proteins was noticed in those with OCS treatment (Figure 3). When comparing samples from PW with (*n* = 111) and without (*n* = 34) OCS to HC, respectively, differential expression of all 10 most differentially expressed proteins was still seen (Figure 3). In terms of the clinical characteristics of PW with and without OCS treatment within 24 h preceding blood sampling, PW with OCS treatment were more often hospitalized at inclusion (87.5% vs. 53.8%, *p* < 0.001 and had lower levels of blood eosinophils (0.05 (0.05–0.05) versus 0.1 (0.05–0.4), *p* < 0.001) than PW without OCS treatment (Table 2).

### 3.5 Protein differences in preschool wheezers at revisit after 3 months

**FIGURE 4:**
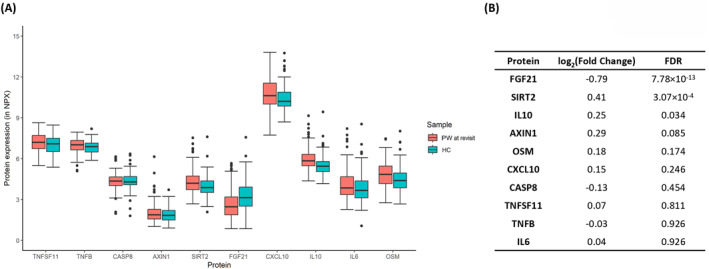
(A) Box plot showing protein expression between children with preschool wheeze at revisit after 3 months (PW‐R) and healthy controls (HC) (B) Fold change and false discovery rate (FDR) of PW‐R in comparison to HC. AXIN1, Axis inhibition protein 1; CASP8, Caspase 8; CXCL10, C‐X‐C motif chemokine ligand 10; FC, fold change; FDR, false discovery rate; FGF21, Fibroblast growth factor 21; HC, healthy controls; IL‐10, Interleukin 10; IL‐6, Interleukin 6; OSM, Oncostatin M; PW‐R, children with preschool wheeze at revisit after 3 months; SIRT2, Sirtuin 2; TNFSF11, TNF superfamily 11; TNFβ, Tumor necrosis factor β.

At revisit after 3 months, three proteins (SIRT2, FGF21 and IL‐10) among the 10 most differentially expressed proteins at inclusion, were still differentially expressed in PW‐R compared to HC. SIRT2 and IL‐10 still had an elevated expression while FGF21 presented with a deceased expression in comparison to HC (Figure 4).

## 4 DISCUSSION

In a group of 145 children recruited during an episode of preschool wheeze (PW), we found significant differences for 52 of the 74 analysed inflammation‐related proteins compared to a group of 101 age‐matched healthy controls (HC). Unsupervised clustering based on the expression of 10 of the 52 proteins separated PW from HC. Expression levels of five of those 10 proteins were significantly associated with OCS intake 24 h preceding blood sampling. However, the difference in protein expression was significant between PW and HC, irrespective of OCS treatment in PW. Of the initially included 145 children, 113 attended a revisit after 3 months (PW‐R). Among the top 10 most differentially expressed proteins, three showed differential expression in PW‐R in comparison to HC, two (IL‐10 and SIRT2) still with elevated expression while one (FGF21) with decreased expression.

The expression profiles of 10 inflammatory proteins separated PW from HC. Seven of these 10 proteins (OSM, IL‐6, IL‐10, CXCL10, SIRT2, FGF21 and AXIN1) were significantly elevated during a wheezing episode, while three showed decreased expression levels compared with HC (TNF‐β, TNFSF11 and CASP8). These 10 proteins are part of the immune response in acute preschool wheezing and are involved in different pathophysiological mechanisms related to the inception of asthma, airway epithelial dysfunction, airway remodelling, impaired antiviral response and T2 inflammation (Table S5).

Our finding of a five‐fold upregulation of OSM in PW compared to HC could be explained by OSMs suggested role in epithelial dysfunction and barrier disruption.^19^, ^20^ The airway epithelium is central for the defence against allergens, pathogens and other environmental factors through structural, mucociliary and immunological barriers, and epithelial dysfunction is important in asthma development.^21^, ^22^ Respiratory viruses, such as RV and RSV reduce the expression of epithelial junction proteins,^21^ indicating the important role of the airway epithelium for the pathophysiological mechanism in preschool wheeze.

Furthermore, we found elevated levels of IL‐6, a pleiotropic cytokine with pro‐inflammatory functions^23^ and also a well‐known contributor to tissue remodelling.^24^ Our findings suggest that the process of airway remodelling is ongoing in children suffering from preschool wheeze, possibly contributing to the increased risk of asthma in these children. Airway remodelling is thought to be a consequence of repeated epithelial injury, poor repair and inflammation.^25^ In children with wheeze, airway remodelling has been shown to be a marker for future asthma risk.^26^

We observed that children with PW compared with HC showed higher plasma levels of IL‐10 (downregulates Th1 cytokines) and CXCL10 (part of the Th1 immune response)^27^, ^28^ and lower levels of TNF‐β, TNFSF11 and CASP8, which have pro‐inflammatory and apoptotic functions due to their activation of NF‐κB signalling pathways.^29^, ^30^, ^31^ High levels of CXCL10 have been correlated with asthma severity, including airflow limitation, as well as predictive of viral‐induced asthma exacerbations,^32^ which is in line with our results. At revisit after 3 months, IL‐10 still showed a higher expression than in HC. Our previous results from the GEWAC cohort demonstrated a skewed IFN‐response in children with wheeze.^33^ Taken together, these findings suggest an exaggerated viral replication that might contribute to the inception of asthma and highlight the importance of the antiviral response in preschool wheeze.

We found elevated levels of IL‐6, SIRT2, FGF21 and AXIN1 in PW in comparison to HC. SIRT2 continued to be upregulated, whereas FGF21 was significantly decreased at revisit after 3 months. T2 inflammation predominates in older children^34^ and contributes to disease progression from preschool wheeze to eosinophilic asthma.^6^ Cytokines derived from T2 inflammation mediate activation of Th2 responses, as shown in our study with elevated levels of IL‐6, which promotes Th2 differentiation,^24^ and SIRT2, which acts pro‐inflammatory by upregulating Th2 responses.^35^ SIRT2 was still upregulated at revisit after 3 months, which could be important for the development of allergic sensitization and asthma.^36^ FGF21 has been shown to correlate with the frequency of circulating mast cells in asthmatic patients^37^, ^38^ and is involved in energy homoeostasis.^37^ Furthermore, FGF21 is a marker that can distinguish controlled from poorly controlled asthma,^39^ which is in line with our findings in PW‐R being in remission. AXIN1 has been reported to correlate with a Th2 high phenotype.^40^ Consequently, these results indicate that markers of T2 inflammation are activated already at an acute wheezing episode in the preschool age.

In addition, we showed that OCS intake within 24 h preceding blood sampling was significantly associated with the protein levels of five of the 10 most differentially expressed proteins: OSM, CASP8, IL‐10, TNF‐β and CXCL10. Corticosteroids alter the balance between pro‐ and anti‐inflammatory proteins and suppress airway inflammation and are suggested to have the best effect in treating symptoms of wheeze in atopic individuals and children with severe wheeze.^41^, ^42^, ^43^ Furthermore, OCS is widely used in emergency departments to relieve symptoms of wheeze, but studies have shown conflicting evidence of efficacy.^44^, ^45^, ^46^ In our study, peripheral blood eosinophil levels were decreased in PW treated with OCS, demonstrating its anti‐inflammatory effect. Nevertheless, the difference in the expression of all 10 proteins was significant between PW and HC irrespective of OCS treatment in PW. Children receiving OCS had a more severe episode of wheeze that demanded hospitalization at inclusion to a higher extent in comparison to those not receiving OCS. However, the clinical data from our cohort did not support a protective effect of OCS treatment with regard to symptom severity after inclusion. Our results show that OCS alters the expression of pro‐ and anti‐inflammatory proteins, but its effect is still questionable.

The major strengths of this study are the analyses of a broad range of inflammatory biomarkers in plasma from a group of preschool children during an acute wheezing episode as well as healthy controls. Another strength is the high‐risk nature of the cohort, with a high prevalence of hospitalization and detailed clinical characterization of preschool wheezers both at inclusion and at the revisit after 3 months. Limitations are that the sample size is not large enough for detailed subgroup analyses, information about events prior to the wheezing episode was reported at the revisit after 3 months, which can imply recall bias, and not all 92 proteins passed the quality check. Finally, not all 145 PWs attended the revisit.

In conclusion, our findings of differential expression of 10 proteins in PW compared with HC provide a framework for understanding the significance of both pro‐ and anti‐inflammatory responses in an acute episode of preschool wheeze. We identified changes in plasma expression of specific proteins previously shown to be involved in different pathophysiological mechanisms of asthma: epithelial dysfunction, remodelling, T2 inflammation and impaired antiviral response. Three of the 10 differentially expressed proteins, involved in T2 inflammation and antiviral response, continued to be differentially expressed at revisit after 3 months, which further supports the presence of common immunopathological pathways between preschool wheeze and asthma.

## AUTHOR CONTRIBUTIONS

**Idun Holmdahl**: Conceptualization (equal); formal analysis (equal); methodology (equal); visualization (equal); writing – original draft (lead). **Sandip Chakraborty**: Formal analysis (equal); Writing – review & editing (equal). **Angela Hoyer**: Writing – review & editing (equal). **Anastasia Filiou**: Writing – review & editing (equal). **Anna Asarnoj**: Writing – review & editing (equal). **Anders Sjölander**: Writing – review & editing (equal). **Magnus P. Borres**: Writing – review & editing (equal). **Marianne van Hage**: Writing – review & editing (equal). **Gunilla Hedlin**: Writing – review & editing (equal). **Jon R. Konradsen**: Conceptualization (equal); writing – review & editing (equal). **Cilla Söderhäll**: Conceptualization (lead); Funding acquisition (lead); methodology (equal); supervision (lead); visualization (equal); writing – review & editing (equal).

## CONFLICT OF INTEREST STATEMENT

I.H., A.F., A.H., S.C. and G.H. have no conflict of interest to report. J.R.K. and C.S. have received non‐financial support from Thermo Fisher Scientific. A.A. has received lecture fees from Thermo Fisher Scientific, ALK, Mylan, Semper, Nestlé and Orion Pharma and advisory board fees from Sanofi, Novartis and Aimmune Therapeutics. A.S. and M.P.B. are employees at Thermo Fisher Scientific. M.v.H. reports lecture fees from Thermo Fisher Scientific outside the submitted work.

## Supporting information

Supporting Information S1

## Data Availability

Due to the nature of this research, participants of this study did not agree for their data to be shared publicly, so supporting data is not available.
