# Supplementary material for: Inflammatory related plasma proteins involved in acute preschool wheeze
Source: Clin Transl Allergy. 2023 Nov 1;13(11):e12308. doi: 10.1002/clt2.12308 (PMC10618892; doi:10.1002/clt2.12308)
Supplement: Supplementary file 1 — Supporting Information S1 [file CLT2-13-e12308-s001.docx]

**Table S1**. Inclusion and exclusion criteria for the GEWAC cohort

|  | Inclusion criteria | Exclusion criteria |
| --- | --- | --- |
| Children with preschool wheeze (cases) | - Age 6-48 months - Presenting at the emergency with acute symptoms of wheeze | - Prematurity (birth before 36 gestational weeks) - Any chronic disease - Any simultaneous complication such as bacterial pneumonia, sepsis, diabetes at the time of inclusion. |
| Control group | - Age 6-48 months | - Prematurity (birth before 36 gestational weeks) - A history of bronchial obstruction/asthma - Known sensitization to airborne allergens |

**Table S2**: List of 92 proteins in the Olink inflammatory panel. 18 proteins had expression below the limit of detection in more than 15% of samples, and were therefore excluded. Rest 74 proteins were included in the analysis.

| **Uniprot Id** | **Gene symbol** | **Used in analysis** |  | **Uniprot Id** | **Gene symbol** | **Used in analysis** |  | **Uniprot Id** | **Gene symbol** | **Used in analysis** |
| --- | --- | --- | --- | --- | --- | --- | --- | --- | --- | --- |
| O95760 | IL33 | No |  | P01138 | NGF | Yes |  | P55773 | CCL23 | Yes |
| P01375 | TNF | No |  | P01374 | TNFB | Yes |  | P78423 | CX3CL1 | Yes |
| P01579 | IFNG | No |  | P01732 | CD8A | Yes |  | P78556 | CCL20 | Yes |
| P01583 | IL1A | No |  | P02778 | CXCL10 | Yes |  | P80075 | MCP2 | Yes |
| P05112 | IL4 | No |  | P03956 | MMP1 | Yes |  | P80098 | MCP3 | Yes |
| P05113 | IL5 | No |  | P05231 | IL6 | Yes |  | P80162 | CXCL6 | Yes |
| P15018 | LIF | No |  | P06127 | CD5 | Yes |  | P80511 | S100A12 | Yes |
| P35225 | IL13 | No |  | P09238 | MMP10 | Yes |  | Q07011 | TNFRSF9 | Yes |
| P50225 | ST1A1 | No |  | P09341 | CXCL1 | Yes |  | Q07325 | CXCL9 | Yes |
| P60568 | IL2 | No |  | P09603 | CSF1 | Yes |  | Q08334 | IL10RB | Yes |
| Q13007 | IL24 | No |  | P10145 | IL8 | Yes |  | Q13261 | IL15RA | Yes |
| Q5T4W7 | ARTN | No |  | P10147 | CCL3 | Yes |  | Q13291 | SLAMF1 | Yes |
| Q8N6P7 | IL22RA1 | No |  | P13232 | IL7 | Yes |  | Q13478 | IL18R1 | Yes |
| Q8NF90 | FGF5 | No |  | P13236 | CCL4 | Yes |  | Q13541 | 4E-BP1 | Yes |
| Q969D9 | TSLP | No |  | P13500 | MCP1 | Yes |  | Q13651 | IL10RA | Yes |
| Q99748 | NRTN | No |  | P13725 | OSM | Yes |  | Q14116 | IL18 | Yes |
| Q9NYY1 | IL20 | No |  | P14210 | HGF | Yes |  | Q14790 | CASP8 | Yes |
| Q9UHF4 | IL20RA | No |  | P14784 | IL2RB | Yes |  | Q16552 | IL17A | Yes |
| O00300 | OPG | Yes |  | P15692 | VEGFA | Yes |  | Q8IXJ6 | SIRT2 | Yes |
| O14625 | CXCL11 | Yes |  | P20783 | NT3 | Yes |  | Q8NFT8 | DNER | Yes |
| O14788 | TNFSF11 | Yes |  | P21583 | SCF | Yes |  | Q8WWJ7 | CD6 | Yes |
| O15169 | AXIN1 | Yes |  | P22301 | IL10 | Yes |  | Q99616 | MCP4 | Yes |
| O15444 | CCL25 | Yes |  | P25942 | CD40 | Yes |  | Q99731 | CCL19 | Yes |
| O43508 | TWEAK | Yes |  | P28325 | CST5 | Yes |  | Q9BZW8 | CD244 | Yes |
| O43557 | TNFSF14 | Yes |  | P29460 | IL12B | Yes |  | Q9GZV9 | FGF23 | Yes |
| O95630 | STAMBP | Yes |  | P39905 | GDNF | Yes |  | Q9H5V8 | CDCP1 | Yes |
| O95750 | FGF19 | Yes |  | P42702 | LIFR | Yes |  | Q9NRJ3 | CCL28 | Yes |
| P00749 | PLAU | Yes |  | P42830 | CXCL5 | Yes |  | Q9NSA1 | FGF21 | Yes |
| P00813 | ADA | Yes |  | P49771 | FLT3LG | Yes |  | Q9NZQ7 | CD274 | Yes |
| P01135 | TGFA | Yes |  | P50591 | TRAIL | Yes |  | Q9P0M4 | IL17C | Yes |
| P01137 | TGFB1 | Yes |  | P51671 | CCL11 | Yes |  |  |  |  |

**Table S3.** Statistical differences between children with preschool wheeze and healthy controls for all 74 proteins included in the analysis.

| **Proteins** | **log_2_FoldChange** | **P-Value** | **FDR** |  | **Proteins** | **log_2_FoldChange** | **P-Value** | **FDR** |
| --- | --- | --- | --- | --- | --- | --- | --- | --- |
| CASP8 | -1.7699 | 1.26×10^−79^ | 3.11×10^-78^ |  | CD8A | 0.0316 | 0.7356 | 0.7776 |
| TNF-B | -1.4595 | 9.47×10^−56^ | 1.00×10^−54^ |  | MMP-1 | 0.0478 | 0.6097 | 0.6539 |
| TNFSF11 | -1.3238 | 2.78×10^−46^ | 2.57×10^−45^ |  | CCL28 | 0.0514 | 0.5873 | 0.6487 |
| TNFRSF9 | -0.9847 | 1.95×10^−26^ | 1.31×10^−25^ |  | LIF-R | 0.0578 | 0.5391 | 0.6045 |
| FIt3L | -0.9565 | 4.87×10^−25^ | 2.77×10^−24^ |  | IL-18 | 0.0735 | 0.4324 | 0.4923 |
| IL-12B | -0.7939 | 1.02×10^−17^ | 5.03×10^−17^ |  | TWEAK | 0.0776 | 0.4073 | 0.4709 |
| CXCL9 | -0.6431 | 3.97×10^−12^ | 1.40×10^−11^ |  | CX3CL1 | 0.0931 | 0.3207 | 0.3767 |
| SLAMF1 | -0.5616 | 2.04×10^−09^ | 6.03×10^−09^ |  | NGF | 0.1045 | 0.2798 | 0.3451 |
| MCP-1 | -0.5594 | 1.62×10^−09^ | 4.98×10^−09^ |  | CDCP1 | 0.1063 | 0.2595 | 0.3254 |
| CCL25 | -0.5451 | 4.31×10^−09^ | 1.23×10^−08^ |  | PD-L1 | 0.1181 | 0.2083 | 0.2658 |
| CCL11 | -0.4846 | 1.82×10^−07^ | 4.80×10^−07^ |  | VEGFA | 0.1705 | 0.0694 | 0.0951 |
| CCL3 | -0.4625 | 6.52×10^−07^ | 1.61×10^−06^ |  | FGF-19 | 0.2127 | 0.0237 | 0.0337 |
| CCL19 | -0.4466 | 1.51×10^−06^ | 3.60×10^−06^ |  | FGF-23 | 0.2311 | 0.0145 | 0.0211 |
| CD6 | -0.4453 | 1.65×10^−06^ | 3.81×10^−06^ |  | MCP-3 | 0.2532 | 8.31×10^−03^ | 0.0128 |
| CCL4 | -0.4300 | 3.73×10^−06^ | 8.37×10^−06^ |  | GDNF | 0.2926 | 2.23×10^−03^ | 0.0037 |
| CD5 | -0.4234 | 5.27×10^−06^ | 1.15×10^−05^ |  | TGFA | 0.3483 | 2.31×10^−04^ | 4.14×10^−04^ |
| IL-10RB | -0.3800 | 4.40×10^−05^ | 9.04×10^−05^ |  | EN-RAGE | 0.3649 | 1.21×10^−04^ | 2.35×10^−04^ |
| IL-15RA | -0.3542 | 2.35×10^−04^ | 4.14×10^−04^ |  | IL-7 | 0.3759 | 8.19×10^−05^ | 1.64×10^−04^ |
| CST5 | -0.3469 | 1.93×10^−04^ | 3.58×10^−04^ |  | IL-18R1 | 0.4011 | 2.17×10^−05^ | 4.59×10^−05^ |
| PLAU | -0.3466 | 1.94×10^−04^ | 3.58×10^−04^ |  | STAMBP | 0.4893 | 2.49×10^−07^ | 6.35×10^−07^ |
| CD244 | -0.3281 | 4.21×10^−04^ | 7.24×10^−04^ |  | IL-8 | 0.5550 | 5.10×10^−09^ | 1.40×10^−08^ |
| TRAIL | -0.3180 | 6.29×10^−04^ | 0.0011 |  | ADA | 0.6092 | 1.56×10^−10^ | 5.01×10^−10^ |
| IL-17A | -0.2629 | 5.11×10^−03^ | 0.0082 |  | CCL23 | 0.6612 | 3.78×10^−12^ | 1.40×10^−11^ |
| MCP-4 | -0.2598 | 5.26×10^−03^ | 0.0083 |  | IL-17C | 0.6634 | 8.21×10^−12^ | 2.76×10^−11^ |
| DNER | -0.2450 | 8.51×10^−03^ | 0.0129 |  | 4×10−BP1 | 0.6683 | 2.26×10^−12^ | 8.81×10^−12^ |
| SCF | -0.2372 | 0.0109 | 0.0161 |  | MMP-10 | 0.6960 | 2.87×10^−13^ | 1.18×10^−12^ |
| CXCL6 | -0.1700 | 0.0683 | 0.0951 |  | MCP-2 | 0.7607 | 1.67×10^−15^ | 7.27×10^−15^ |
| IL-2RB | -0.1619 | 0.0888 | 0.1195 |  | HGF | 0.7619 | 1.51×10^−15^ | 6.97×10^−15^ |
| OPG | -0.1365 | 0.1434 | 0.1895 |  | CXCL11 | 0.8400 | 1.78×10^−18^ | 9.41×10^−18^ |
| CCL20 | -0.1235 | 0.1859 | 0.2413 |  | TNFSF14 | 1.0101 | 1.47×10^−25^ | 9.07×10^−25^ |
| CXCL5 | -0.0994 | 0.2871 | 0.3483 |  | CXCL10 | 1.1383 | 6.71×10^−32^ | 4.96×10^−31^ |
| CD40 | -0.0933 | 0.3178 | 0.3767 |  | SIRT2 | 1.1587 | 9.54×10^−33^ | 7.84×10^−32^ |
| NT-3 | -0.0496 | 0.5985 | 0.6513 |  | AXIN1 | 1.5745 | 5.32×10^−56^ | 6.56×10^−55^ |
| TGFB1 | 0.0168 | 0.8572 | 0.8572 |  | FGF-21 | 1.6342 | 5.79×10^−61^ | 8.57×10^−60^ |
| IL-10RA | 0.0211 | 0.8260 | 0.8373 |  | IL-6 | 1.7470 | 6.70×10^−69^ | 1.24×10^−67^ |
| CSF-1 | 0.0235 | 0.8019 | 0.8242 |  | IL-10 | 1.9963 | 1.43×10^−87^ | 5.31×10^−86^ |
| CXCL1 | 0.0284 | 0.7619 | 0.7941 |  | OSM | 2.3823 | 3.38×10^−119^ | 2.50×10^−117^ |

**Table S4.** Drop-out analysis of children with preschool wheeze (PW) not attending the revisit**.

| Variables | PW not attending revisit n = 32 | PW attending revisit n = 113 | p-value |
| --- | --- | --- | --- |
| Age in months, median (IQR) | 18.5 (13-25) | 17 (12-24.5) | 0.74 |
| Male sex, n (%) | 22 (68.8) | 75 (66.4) | 0.80 |
| Caucasian mother and/or father, n (%) | 21 (95.5) | 100 (88.5) | 0.47 |
| Maternal smoking during pregnancy, n (%) | 0 (0) | 14 (12.4) | 0.13 |
| Exclusive breastfeeding 4 months, n (%) | 15 (71.4) | 65 (59.1) | 0.29 |
| Parental asthma/allergy, n (%) | 15 (68.2) | 83 (74.1) | 0.57 |
| >6 RTIs/year, n (%) | 12 (54.5) | 73 (66.4) | 0.29 |
| Previous RSV infection, n (%) | 9 (40.9) | 31 (27.4) | 0.21 |
| Attend childcare or family home day-care, n (%) | 14 (87.5) | 79 (69.9) | 0.23 |
| LTRA at inclusion, n (%) | 2 (6.3) | 11 (9.7) | 0.73 |
| ICS at inclusion, n (%) | 13 (40.6) | 46 (40.7) | 0.99 |
| OCS within 24 h of blood sampling, n (%) | 21 (65.6) | 85 (75.2) | 0.28 |
| Reported food allergy at inclusion, n (%) | 1 (4.5) | 12 (10.7) | 0.69 |
| Eczema at inclusion, n (%) | 3 (13.6) | 23 (20.4) | 0.57 |
| First time wheeze, n (%) | 1 (11.1) | 26 (23.0) | 0.68 |
| Hospitalized at inclusion, n (%) | 6 (66.7) | 92 (81.4) | 0.38 |
| RTIs at inclusion*, n (%) | 29 (90.6) | 108 (95.6) | 0.38 |
| Doctor's diagnosis of asthma at inclusion, n (%) | 19 (59.4) | 60 (53.1) | 0.53 |
| Eosinophils at inclusion, median (IQR) | 0.05 (0.05-0.05) | 0.05 (0.05-0.1) | 0.49 |
| Neutrophils at inclusion, median (IQR) | 8.0 (3.9-12.8) | 7.1 (4.4-10.3) | 0.17 |

* All 8 who answered "No" had positive NPH samples (3 Bocavirus, 1 Parainfluensa, 3 Rhinovirus and 1 Respiratory Syncytial Virus) ** Plasma not available at revisit

**Table S5.** Basic information about the top ten differentially expressed proteins between PW and HC.

| Protein expression^1^ | Protein name | Abbreviation | Function |
| --- | --- | --- | --- |
|  | TNF superfamily member 11 | TNFSF11 | TNF superfamily (TNFSF) exhibits pro-inflammatory functions and can also trigger apoptosis, partially due to their activation of NF-κB signalling pathways (21). TNF-β and TNFSF11 are both ligands of the TNFSF. NF-κB control many genes involved in inflammation and immune regulation as well as regulating cell proliferation, apoptosis and differentiation (29, 30). |
|  | Tumor necrosis factor beta | TNF-β | See above. |
|  | Caspase 8 | CASP8 | A pathway induced by NF-κB involves CASP8. Activation of CASP8 induce an apoptotic response, however some viruses have the ability to inhibit CASP8 and therefore prevent apoptosis resulting in enhanced viral replication. (31). |
|  | Axis inhibition protein 1 | AXIN1 | Wingless/integrase-1 (WNT) signalling pathway is an important gene in controlling lung development and WNT signalling has been found to associate with asthma risk, AXIN1 acts a negative regulator in this pathway. A Th2-high phenotype is associated with upregulated WNT-negative regulators (40). |
|  | Sirtuin 2 | SIRT2 | SIRT2, a histone deacetylase, acts pro inflammatory by upregulation of Th2 responses, blocking of SIRT2 has shown to reduce airway inflammation and symptoms of asthma, suggesting that the increase of SIRT2 may contribute to the pathogenesis of airway inflammation (35) |
|  | Fibroblast growth factor 21 | FGF21 | FGF21 is a soluble protein that has been correlated with circulating mast cell frequency among allergic asthmatic patients as well as reduced FEV1 (37-39). |
|  | C-X-C motif chemokine ligand 10 | CXCL10 | Concentrations of CXCL10 are elevated in virus- or RV-induced asthma and is a known chemoattractant for Th1 cells which acts to eliminate viral pathogens, although when uncontrolled can lead to pathology (28). CXCL10 can be secreted by multiple cell types, including bronchial epithelial cells and high levels correlates with disease severity, including airflow limitation (32). |
|  | Interleukin 10 | IL10 | IL-10 is a potent anti-inflammatory cytokine, produced by a wide variety of immune cells, which inhibits the synthesis of many inflammatory proteins (27). |
|  | Interleukin 6 | IL6 | IL-6 is a pleiotropic cytokine with well-known pro-inflammatory functions, it stimulates the production of acute phase proteins from the liver and induces acute phase responses (23). IL-6 promotes differentiation of Th2 cells as well as acting as a regulator in Th17 immune responses, inducing neutrophilic inflammation and it also contributes to tissue remodelling (24). |
|  | Oncostatin M | OSM | OSM is associated with barrier disruption in the airway epithelium and levels of OSM in nasal tissue and bronchoalveolar lavage fluid (BAL) among allergic asthmatics are correlated with markers of epithelial dysfunction (19, 20). |

^1^ Higher ( ) or lower ( ) protein expression in comparison to healthy controls (HC)

**Table S6.** Baseline characteristics of children with an acute episode of preschool wheeze (PW) that cluster with healthy controls (HC) in comparison to the rest of the PW samples.

| **Variables** | **PW among HC n = 9** | **PW n = 136** | **p-value** |
| --- | --- | --- | --- |
| Age in months, median (IQR) | 11 (9-19) | 18 (12-25) | 0.093 |
| Male sex, n (%) | 4 (44.4) | 93 (68.4) | 0.14 |
| Caucasian mother and/or father, n (%) | 8 (100) | 14 (89) | 1 |
| Maternal smoking during pregnancy, n (%) | 1 (12.5) | 13 (10.2) | 0.59 |
| Exclusive breastfeeding 4 months, n (%) | 5 (71.4) | 75 (60.5) | 0.71 |
| Parental asthma/allergy, n (%) | 6 (75) | 92 (73) | 1 |
| >6 RTIs^1^/year, n (%) | 4 (50) | 81 (65.3) | 0.45 |
| Previous RSV^2^ infection, n (%) | 3 (37.5) | 37 (29.1) | 0.69 |
| Attend childcare or family home day-care, n (%) | 6 (75) | 87 (71.9) | 1 |
| LTRA^3^ at inclusion, n (%) | 0 | 13 (9.6) | 1 |
| ICS^4^ at inclusion, n (%) | 2 (22.2) | 57 (41.9) | 0.31 |
| OCS^5^ within 24 h before blood sampling, n (%) | 7 (77.8) | 104 (76.5) | 1 |
| Reported food allergy at inclusion, n (%) | 1 (12.5) | 12 (9.5) | 0.57 |
| Fx5 positive, n (%) | 2 (25) | 25 (22.9) | 1 |
| Phadiatop positive, n (%) | 0 | 9 (8.3) | 1 |
| Atopic dermatitis at inclusion, n (%) | 1 (12.5) | 25 (19.7) | 1 |
| Vitamin D nmol/L, median (IQR) | 85.5 (81-116.3) | 80 (68-101) | 0.24 |
| First time wheeze, n (%) | 3 (37.5) | 24 (21.1) | 0.37 |
| Hospitalized at inclusion, n (%) | 6 (75) | 92 (80.7) | 0.66 |
| RTI^1^* at inclusion, n (%) | 7 (77.8) | 130 (95.6) | 0.079 |
| Doctor's diagnosis of asthma at inclusion n (%) | 4 (44.4) | 75 (55.1) | 0.73 |
| Doctor's diagnosis of asthma at the revisit, n (%) | 4 (50) | 60 (52.6) | 1 |
| **Eosinophils at inclusion 10^9^/L, median (IQR)** | 0.2 (0.075-0.75) | 0.05 (0.05-0.075) | **0.001** |
| **Neutrophils at inclusion 10^9^/L, median (IQR)** | 3.7 (2.5-6.6) | 7.7 (4.5-10.7) | **0.007** |
| Eosinophils at the revisit 10^9^/L, median (IQR) | 0.6 (0.3-0.9) | 0.3 (0.2-0.6) | 0.20 |
| Neutrophils at the revisit 10^9^/L, median (IQR) | 2.9 (2.1-3.6) | 3.2 (2.2-4.3) | 0.67 |
| Rhinovirus, n (%) | 3 (33.3) | 58 (43.3) | 0.73 |
| RSV^2^, n (%) | 2 (22.2) | 24 (17.9) | 0.67 |

^1^Respiratory tract infections, ^2^Respiratory Syncytial Virus, ^3^Leukotriene Receptor Antagonist, ^4^Inhaled Corticosteroids, ^5^Oral Corticosteroids, * All 8 who answered "No" had positive NPH samples (3 Bocavirus, 1 Parainfluenza, 3 Rhinovirus and 1 Respiratory Syncytial Virus)
